# The human health benefits from GM crops

**DOI:** 10.1111/pbi.13261

**Published:** 2019-10-02

**Authors:** Stuart J. Smyth

**Affiliations:** ^1^ Department of Agricultural and Resource Economics College of Agriculture and Bioresources University of Saskatchewan Saskatoon Saskatchewan Canada

**Keywords:** cancer, farmer suicide, nutrition, pesticide poisonings, reductions in chemical use

Genetically modified (GM) crops represent the most rapidly adopted technology in the history of agriculture, having now reached 25 years of commercial production. Grown by millions of farmers, many in developing countries, the technology is providing significant economic and environmental benefits, such as reductions in chemical use of 37%, increased yields of 22% and improved farm profits of 68% (Klümper and Qaim, 2014). While knowledge and awareness of these benefits are increasingly communicated, less well known are the benefits that GM crops are providing to humans and human health.

From an adoption percentage, countries with highly industrialized, large‐scale agricultural production are the significant benefactors. Production in Argentina, Australia, Brazil, Canada and the USA accounts for the majority of global GM crop acreage, with farmers in these five countries capturing the majority of the economic and environmental benefits. Conversely, because of the lack of mechanized agricultural production, it is the small landholder farmers in developing countries that accrue the majority of the human health benefits.

## Reductions in pesticide poisonings

While the application of agricultural chemicals is highly mechanized in industrial countries, the same cannot be said for developing countries, where most applications are done through human labour using handheld applicators. With average farm sizes commonly <10 acres in many developing countries, field sizes are even smaller, resulting in much, if not all, of the fieldwork (seeding, weeding, spraying and harvesting), being done by hand. Chemical applications, especially insecticides on crops such as cotton and brinjal, require numerous applications throughout the course of the growing season to ensure insect damage is as minimal as possible. This is crucial with brinjal production as visual evidence of insect damage prevents the sale of products for public consumption, resulting in significant income losses. The application of insecticides must be done as the plants grow and mature, through the use of backpack sprayers, resulting in skin absorption of chemical residues. Exposures to chemicals such as this result in sickness of the person applying the chemicals, known as pesticide poisoning. GM crops, particularly Bt cotton, have resulted in significant reductions in pesticide poisoning cases due to reduced applications and reduced levels of insecticide exposure.

Reductions in farmer pesticide poisonings have been quantified in China, India, Pakistan and South Africa. Often, cases of pesticide poisoning are not formally reported to health centres and the results on pesticide poisoning may be underestimated due to the lack of reporting. In South Africa, farmers reduced pesticide applications from 11.2 per year to 3.8, with reported cases of pesticide poisoning declining from over 50 per year to <10 over the first 4 years of Bt cotton adoption (Bennet *et al*., 2003). One third of non‐Bt cotton farmers in China reported cases of pesticide poisoning, compared with 9% of Bt cotton‐producing farmers (Hossain *et al*., 2004). Assessing the health impacts in India reveals a reduction in cases of pesticide poisoning of 2.4–9 million cases per year (Kouser and Qaim, 2011). Cumulatively, since 2003, when Bt cotton was first commercialized in India, a minimum of 38 million fewer instances of pesticide poisoning have occurred, with an upper potential of 144 million. Farmers in Pakistan growing non‐Bt cotton reported up to seven instances of pesticide poisoning in the growing season with 35% reporting no instances, versus Bt cotton farmers reporting up to six poisonings with 45% reporting none (Kouser and Qaim, 2013).

A medical assessment of 246 Chinese farmers, involving 35 health indicators, found that fungicides associated with the production of non‐Bt cotton had linkages to damaged liver function, while the insecticides used in non‐Bt cotton production may be associated with severe nerve damage (Zhang *et al*., 2016). The use of non‐glyphosate tolerant crops was found to likely reduce renal function and decrease serum folic acid.

## Changes in farmer suicide

Mental health challenges and issues affect all walks of life and economic sectors, with agriculture being no different. Access to sufficient mental health resources can be problematic within the agriculture sector due to rural areas, remote locations and lack of access to mental health support systems. Unfortunately, suicide is a concern in agriculture. India has one of the highest suicide rates in the world, and research has examined the relationship between farmer suicide and the adoption of GM cotton.

Research examining the relationship between farm suicide and Bt cotton adoption revealed a plateauing of the suicide rate following the commercialization of Bt cotton (Gruère and Sengupta, 2011). Farmer suicides were trending upward from 15 000 per year, peaking in 2004, the year after Bt cotton was first commercialized in India. By 2007, the actual suicide rate was 25% below the extrapolated suicide rate. Cumulatively, the reduced rate of suicide associated with the adoption of Bt cotton represents the prevention of a minimum of 75 000 farmer suicides.

## Lowering cancer incidences

The development of insect‐resistant crop varieties has begun to have a noticeable potential to improve human health through the reduction in cancer rates. Prior to the commercialization of Bt crops, maize in particular, insect damage to the harvested crop increased the potential for the development of harmful health effects. A study of 21 years of maize production quantified that Bt maize contained lower concentrations of mycotoxins (29%), fumonisins (31%) and thricotecens (37%) (Pellegrino *et al*., 2018). Mycotoxins are both toxic and carcinogenic to humans and animals and are considerably more concerning in developing economy food systems where access to food safety toxicity tests is less prevalent. Fumonisins are correlated to being the cause of higher rates of neural tube defects in high maize‐based diets (Missmer *et al*., 2006). With food security challenges existing in many developing countries, corn containing mycotoxins are consumed as part of the household diet due to the lack of any other option.

## Mental health benefits

One factor not assessed to date is the mental health improvements incurred by GM crop adopters. Stress in agriculture is like every other sector of the business economy, although in the agriculture sector, the stresses may be more related to financial debt servicing and the potentials of crop failure. Both of these factors can contribute to the stress burden of farmers. With the quantified higher yields from GM crops (Klümper and Qaim, 2014), farmers can now gain some degree of confidence that their crop will not fail due to insect pressures, be overcome with weeds and be more resilient should a drought occur.

## Nutritional benefits

Genetically modified crops have made significant contributions to address the United Nations Sustainable Development Goals, in particular goals 1 (reducing poverty) and 2 (reducing hunger). While increased yields have contributed to higher household incomes, which reduce poverty, the increased yields have also enhanced household food security. Biofortified GM crops have been adopted, increasing micronutrient availability (Hefferon, 2014). Nutritionally enhanced foods improve an individual's nutrient intake, preventing and/or treating leading causes of death such as cancer, diabetes, cardiovascular disease and hypertension. Improving the nutritional content of daily food consumption certainly has day‐to‐day effects, but of significant importance are the long‐term effects that extend for decades over the course of an individual's lifetime.

In many instances, improving macronutrients (proteins, carbohydrates, lipids, fibre) and micronutrients (vitamins, minerals, functional metabolites) has significant childhood health improvements, such as reducing blindness due to the lack of vitamin availability. Improved food nutrient content, especially the increase in mineral availability, contributes to improved immunity systems and reduces stunting. In many developing countries, plant‐based nutrient intake accounts for one hundred per cent of an individual's nutrient diet, further highlighting the importance of nutritionally enhanced crop‐derived foods. As the later in life benefits from improved childhood nutrition are better understood, the full value of nutritionally enhanced GM crops and foods may not be realized for several decades.

## Concluding remarks

While millions of farmers growing Bt cotton are experiencing reduced incidences of pesticide poisoning, all of the estimated 17 million farmers growing GM crops globally have reduced chemical exposures. Certainly, the reduced rates of pesticide poisoning, possibly in excess of 100 million cases, are a vital statistic of the benefits of GM crops, but perhaps the most significant is the contribution to improved mental health from farmers, especially those in India. Suicide is a devastating part of agriculture, to which no country is immune and the observed plateauing and now reduction in Indian farmer suicide rates is a benefit that simply cannot be surpassed. By allowing cotton farmers to be more profitable, Bt cotton has allowed tens of thousands of Indian cotton farmers to have more options and opportunities to continue farming. The true benefit of GM crops can be measured through the thousands of family members who no longer have to deal with the anguish and grief suicide causes.

Ongoing mental health improvements from the reduced stress of the potential for crop failure and the damaging effects this has on profitability and food security, while significantly difficult to measure, will continue to be one of the exceptional, but silent, benefits from GM crop production.

## Conflict of Interest

I declare no conflict of interest.
